# Age at Mortality in Patients with Type 2 Diabetes Who Underwent Kidney Transplantation: An Analysis of Data from the Korean National Health Insurance and Statistical Information Service, 2006 to 2018

**DOI:** 10.3390/jcm12093160

**Published:** 2023-04-27

**Authors:** Sun Ok Song, Eugene Han, Kang Ju Son, Bong-Soo Cha, Byung-Wan Lee

**Affiliations:** 1 Divison of Endocrinology, Department of Internal Medicine, National Health Insurance Service Ilsan Hospital, Goyang 10444, Republic of Korea; 2 Divison of Endocrinology, Department of Internal Medicine, Keimyung University School of Medicine, Daegu 42601, Republic of Korea; 3 Research and Analysis Team, National Health Insurance Service Ilsan Hospital, Goyang 10444, Republic of Korea; 4 Division of Endocrinology, Department of Internal Medicine, Yonsei University College of Medicine, Seoul 03722, Republic of Korea

**Keywords:** mortality, diabetes mellitus, end-stage renal disease, kidney transplantation, epidemiology

## Abstract

Background: Although the clinical outcomes of diabetes have improved, diabetes remains the principal cause of end-stage renal disease. The aim of the study is to investigate whether mortality trends in individuals with type 2 diabetes and kidney transplantation (KT) have changed. Methods: This study analyzed data from the National Health Insurance Service claims database linked to death records from the National Statistical Information Service in Korea. Information from a total of 2521 deaths of KT recipients was collected from 2006 to 2018. Results: The age at death of KT recipients increased from 57.4 years in 2006 to 65.2 years in 2018, with a mean change of +0.65 years/year (*p* < 0.001). The overall age at death increased by 0.55 and 0.66 years/year in the type 2 diabetes and non-diabetes populations, respectively. The age at death was significantly higher in the type 2 diabetes group, and was maintained during the study period. The proportion of death due to malignancy and cerebrovascular and heart disease was maintained, that due to type 2 diabetes decreased and that due to pneumonia increased. Neither diabetes nor hypertension determined the age at death, and the age at KT was the most prominent factor affecting age at death in KT recipients. Conclusions: The age at death in KT recipients increased over the 12 years between 2006 and 2018, with similar trends in the type 2 diabetes and non-diabetes groups. The age at KT was higher in patients with type 2 diabetes, and was the main contributor to the age at death in KT recipients.

## 1. Introduction

Diabetes mellitus is one of the principal underlying etiologies of end-stage renal disease (ESRD). It eventually requires renal replacement therapy (RRT), including hemodialysis, peritoneal dialysis, and/or kidney transplantation (KT) [1]. Recently, new renoprotective antihypoglycemic agents, sodium-glucose cotransporter 2 inhibitors, and glucagon-like peptide-1 receptor agonists have been developed [2]. However, the incidence of ESRD due to type 2 diabetes mellitus (T2DM) is expected to increase with the increased prevalence of obesity and the aging population. Globally, the prevalence of ESRD has increased from 19.0% in 2000 to 29.7% in 2015 among individuals with diabetes. The incidence of RRT has increased from 22.1% in 2000 to 31.3% in 2015 [1]. In Korea, the total number of patients with diabetes and ESRD increased steadily and has plateaued since 2012 [3]. Despite a slight decrease in the incidence of ESRD with diabetes over the last decade, a steady increase in ESRD in the elderly population (over 75 years) has contributed to the overall healthcare burden in Korea [4].

Among RRTs, KT has shown the best outcomes, including survival and mortality rates [5]. In addition, the clinical prognosis of KT has improved over the past years [6]. However, KT recipients remain at an increased risk of malignancy, atherosclerotic cardiovascular disease (ASCVD), and infection vulnerability due to immunosuppressant treatment [7]. ASCVD is the main cause of death in individuals with ESRD and KT [8]. The high incidence of ASCVD mortality in patients with KT likely stems from multiple underlying cardiometabolic risk factors including diabetes, hypertension, and dyslipidemia, which may have developed before or after transplantation, resulting in a cumulative burden on the heart and vessels.

Recently, several studies have shown improved mortality outcomes in patients with diabetes [9,10]. We also reported an increase in age at death in a population with diabetes. This was mainly due to a decline in death from ASCVD after controlling for dyslipidemia and hypertension [10]. However, it remains unknown whether this improvement in mortality is relevant to KT recipients with diabetes. Thus, we investigated recent mortality trends in patients with diabetes undergoing KT by comparing the cause of death in KT recipients with and without diabetes. This retrospective study used data from the National Health Insurance database maintained by the Korean National Health Insurance Service (NHIS) with mortality records from the National Statistical Information Service (NSIS).

## 2. Methods

### 2.1. Study Subjects

In this nationwide cohort study, we analyzed the data of the insured population obtained from the Korean NHIS claims database (NHIS-2022-1-513). Details of the Korean NHIS claim database have been previously reported [11]. The NHIS is a mandatory insurance organization in Korea that governs the enrollment of the insured and their dependents, gathers contributions, and handles medical fee payment schedules [11]. After de-identification, the Korean NHIS provides claims data containing birth date, region of residence, sex, and hospital visit dates. The data of deceased insured patients from 1 January 2006, to 31 December 2018 was collected. To obtain information on age at death, we followed a cohort of 3,394,502 deceased insured patients and requested the NSIS to provide their records and cause of death. The study was reviewed and authorized by the Institutional Review Board of the National Health Insurance Service Ilsan Hospital (NHIMC-2021-09-025).

### 2.2. Confirmation of Diabetes and Death Certificates

Individual medical records from the 2 years before the date of death were analyzed. The patients was considered to have T2DM if the following three criteria were met: (1) had a T2DM diagnosis code (principal or additional code), (2) had at least one service claim with a diagnosis of T2DM (in outpatient or inpatient care), and (3) received more than one prescription of antidiabetic medication. Principal and additional diagnoses were established using the International Classification of Diseases, 10th revision (ICD-10) codes for T2DM (E11–E14). We extracted the data of all patients who underwent KT from the NHIS database between 2006 to 2018 as defined by the following three criteria: the ICD-10-CM code Z94.0 (KT state), electronic data interchange codes for all claims with R3280 (KT) and R3170 (ureterosigmoidostomy), and benefit extension policy (BEP) application codes with V005. In Korea, BEP helps to support the medical expenses of patients with rare and incurable diseases such as cancer and rare genetic disorders which are associated with a high economic burden [12]. As BEP can provide substantial financial support for treatment, these patients are highly likely to be registered for BEP. Similarly, the patients in the untreated group were likely not treated with KT. Hypertension was characterized using the corresponding ICD-10 codes (I10–13, I15) with treatment with antihypertensive medications.

To determine the age at death and cause of death, we obtained the NSIS death records with disease-specific codes. We extracted the five most prevalent causes of death in South Korea: malignancy (C00–C97), cerebrovascular disease (I60–I69), heart disease (I20–I25), T2DM (E11–E14), and pneumonia (J09–J18). First, the underlying causes of death specified in the death certificates according to the ICD by the World Health Organization were determined. The causes of death were also determined in accordance with the Korean standard classification of disease and case of death [13,14]. To analyze the trends in the cause of death, we divided the study period into the following intervals; 2006–2010, 2011–2014, and 2015–2018.

### 2.3. Statistical Analysis

The results of continuous variables are expressed as means ± standard deviations (SD), and categorical variables are described as the number and frequency percentage (%). Numerical and frequency data were analyzed using a t-test and chi-square test, respectively. Individuals were categorized into two groups based on the presence or absence of diabetes. Two-way analysis of variance with type III sum of squares was applied to determine the significance of differences by groups. Generalized linear model analysis was performed to confirm the factors contributing to age at death, with sex, T2DM, hypertension, age at KT, and years from KT to death as explanatory variables and age at death as the dependent variable. A *p*-value < 0.05 was considered statistically significant. All analyses were conducted using the statistical software SAS version 9.4 (SAS Institute, Cary, NC, USA).

## 3. Results

**Figure 1:**
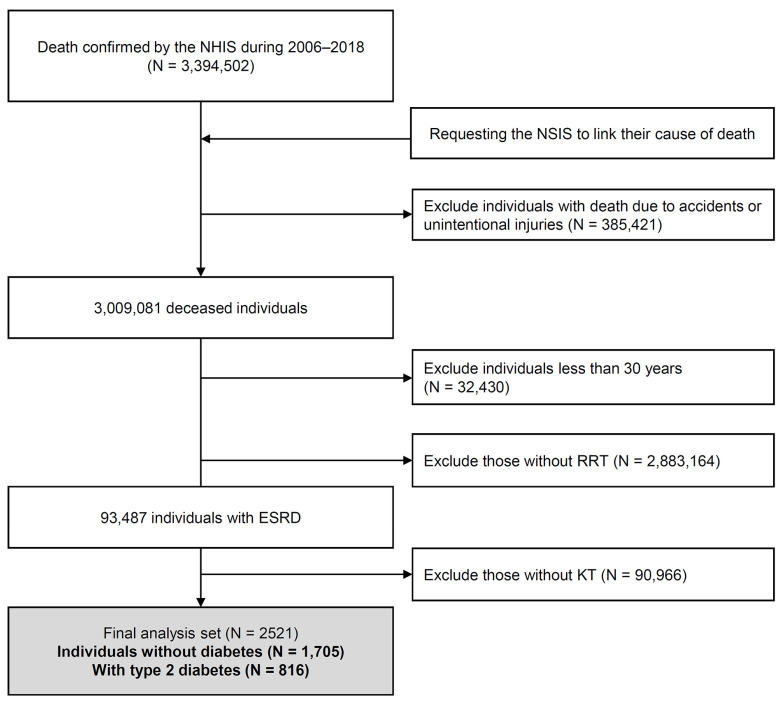
Flow diagram of subject inclusion and exclusion in the Korean National Health Insurance Service claim database and National Statistical Information Service. NHIS—Korean National Health Insurance Service; NSIS—National Statistical Information Service; RRT—renal replacement therapy; ESRD—end-stage renal disease; KT—kidney transplantation.

According to the NHIS database, 3,394,502 individuals died between 2006 and 2018. Of them, those dying due to accidental or unintentional injuries (385,421) were excluded (Figure 1). We selected 2,976,651 individuals aged ≥ 30 years and excluded 2,974,130 of them who met the following criteria: (1) individuals without RRT (*n* = 2,883,164) and (2) those who received RRT other than KT (*n* = 79,792 for hemodialysis, and *n* = 11,174 for peritoneal dialysis). Thus, 2521 subjects who underwent KT (1705 individuals without diabetes and 816 individuals with T2DM) were included in the final analysis.

The characteristics of the study population are shown in Table 1. The age at death was higher in the T2DM group than in the non-diabetes group (65.3 vs. 61.5, *p* < 0.0001), and the proportion of individuals who died at >70 years of age was higher in the T2DM group (35.3% vs. 27.3%, *p* < 0.001). The prevalence of hypertension was higher in the diabetes group (86.5% vs. 76.5%, *p* < 0.001). The mean age at KT was 54.7 years in the non-diabetes and 58.3 years in the T2DM group (*p* < 0.001). However, the years from KT to death were comparable between both groups. There was no significant difference in the proportion of medical aids recipients between the T2DM and non-diabetes groups (16.3% vs. 19.9%, *p* = 0.300).

**Figure 2:**
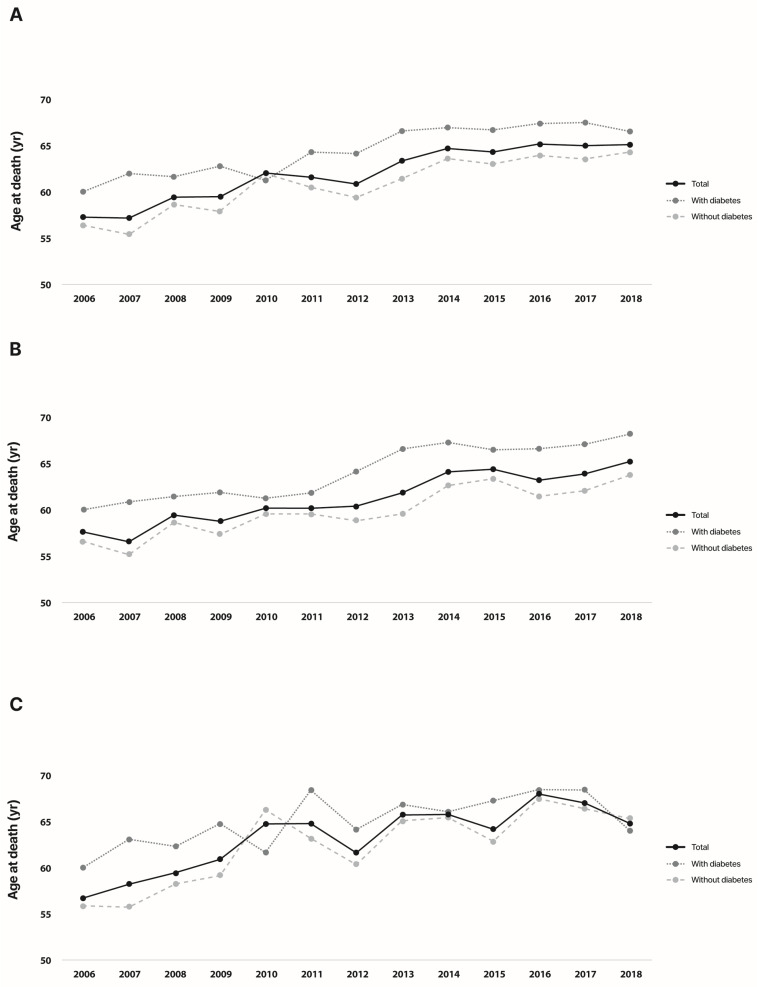
Changes in age of death among individuals with type 2 diabetes and without diabetes. (**A**) overall population, (**B**) men, (**C**) women. The black solid lines represent in total population; grey dot line, a group with type 2 diabetes; light grey dot line, a group without diabetes.

We analyzed the age at death every year during the study period to identify the trend change in age at death. The age at death among KT recipients increased from 57.4 years in 2006 to 65.2 years in 2018, and the mean change was +0.65 years/year (*p* < 0.001) (Figure 2). In the T2DM group, the age at death increased from 60.1 years to 65.2 years, and the annual change in the age at death was 0.54 years/year (*p* < 0.001). Individuals with T2DM were older than those without diabetes at the time of death by an average of 3.67 years (*p* < 0.001). In 2006, the mean age at death in the non-diabetes group was 56.4 years. It increased by 0.66 years/year over 12 years and reached 64.3 years in 2018. The most common cause of death in our study population was malignancy (*n* = 573, 22.7%), followed by cerebrovascular and heart disease (*n* = 275, 10.9%), T2DM (*n* = 247, 9.8%), and pneumonia (*n* = 88, 3.2%). Other miscellaneous causes of death included diseases related to the genitourinary tract (*n* = 596, 23.6%) and, gastrointestinal tract (*n* = 170, 6.7%), circulatory diseases other than cerebrovascular or heart disease (*n* = 160, 6.3%), infectious diseases other than pneumonia (*n* = 136, 5.4%), and diseases related to the respiratory system other than pneumonia (*n* = 67, 2.7%). The proportion of death from malignancy was higher in the non-diabetes group (24.2% vs. 19.7%), while the proportions of death due to cerebrovascular and heart disease (12.9% vs. 10.9%), and T2DM (17.3% vs. 6.2%) were higher in T2DM group (*p* < 0.005). There was no significant difference in the proportion of death due to pneumonia or other miscellaneous causes between T2DM and the non-diabetes groups.

Similar trends were observed when the participants were divided by sex. Age at death was increased by 0.64 and 0.66 years/year among men and women, respectively. Among men, the annual change in the age at death was 0.69 years/year in the T2DM group, which was greater than that in the non-diabetes group (0.59 years/year). Among women, KT recipients with T2DM died at an older age than their counterparts without diabetes over the study period, except in 2010. However, the annual change in the age at death was greater in the non-diabetes group than in the T2DM group (0.77 years/year vs. 0.32 years/year), similar to that in 2018 (64.1 years and 65.2 years in the T2DM and the non-diabetes group, respectively). T2DM status did not affect the change in the age at death in any group (*p* = 0.659 for the overall population, *p* = 0.925 for men, and *p* = 0.462 for women).

The cause of death among KT recipients also changed during the 12-year study period. Overall, the proportion of death due to malignancy accounted for 22.7% of all deaths. This level was maintained throughout the study period (23.3% in 2006–2010 to 22.2% in 2015–2018). Death from cerebrovascular and heart diseases contributed to 10.9% of total deaths. This rate plateaued during the study period (11.1% in 2006–2010 to 10.1% in 2015–2018, Figure 3). However, the proportion of deaths due to pneumonia increased from 1.9% in 2006–2010 to 5.0% in 2015–2018. This trend was also observed in the non-diabetes group. Among KT recipients with T2DM, the overall deaths from malignancy, diabetes, and cerebrovascular and heart disease accounted for 19.7%, 17.3%, and 12.9% of deaths, respectively. Death from malignancy decreased slightly from 23.6% to 19.3%, and death caused by cerebrovascular and heart diseases remained steady, from 12.0% to 12.7%. The proportion of deaths caused by pneumonia increased steeply from 1.9% to 5.2% during the study period.

We analyzed the factors contributing to age at death in KT recipients (Table 2). T2DM and hypertension did not affect the age at death. However, the age at KT was the most prominent factor affecting the age at death in our dataset (β coefficient = 0.99, *p* < 0.001) followed by years from KT to death (β coefficient = 0.97, *p* < 0.001). Female sex was closely related to the age at death; however, the effect of this factor was low (β coefficient = 0.04, *p* < 0.001 for females).

Findings of the generalized linear model for the variables significantly associated with age at death. KT—kidney transplantation.

## 4. Discussion

With the increased prevalence of obesity and an overall aging population, diabetes and chronic kidney disease have become a major socioeconomic problem [15]. Diabetes is a harmful condition that worsens the outcome in KT recipients. In addition, prediabetes can contribute to the cardiovascular burden after KT [16,17]. Based on a previous report demonstrating improvement in age at death in a population with diabetes [10], we focused on the improvement in mortality among patients with T2DM who had undergone KT. We combined data from the NHIS and NSIS databases reflecting national representative populations.

This study had three major findings. First, the age at death of KT recipients in the total population increased significantly from 57.4 years in 2006 to 65.2 years in 2018. Second, during the 12-year follow-up, individuals without diabetes died younger than those with T2DM after KT (*p* < 0.0001). Third, the causes of death among patients with T2DM who undergo KT have changed.

The mean age at death of KT recipients increased by 0.65 years/year from 2006 to 2018. For the general Korean population, we previously reported that during the 12-year study period, the overall age at death increased by 0.44 and 0.26 years/year the diabetes and metabolically healthy control populations, respectively [10]. In this study, during the 12-year study period, the overall age at death among KT recipients increased by 0.54 and 0.66 years/year in the T2DM and non-diabetes groups, respectively. The trend of increased mortality with age has been steady, especially among men with KT, except in 2010 and 2018. Since the first KT case in Korea in 1969, the number of KT cases has gradually increased, reaching 19.4% of the total RRT population in 2019 [3]. Although the prevalence of ESRD in Korea has increased [3], its mortality rate has remained stable in the recent decade [4]. Considering the increased mean age at KT intervention [3], and the stabilized mortality rate of the ESRD population, our results align with previous findings reflecting the increase in KTs in the elderly population and the improvement of lifespan in the KT population.

Considering the higher age at death in the T2DM group than in non-diabetes group, the disparity in the mean age at death between both groups was 3.7 years. Additionally, this difference was maintained throughout the study period, except in 2010 (*p* < 0.0001). The higher age at death in patients with T2DM who underwent KT might be attributable to the age at KT and improvement in post-KT care. In Korea, the mean age in initiation of RRT is higher in patients with diabetes than those with other diseases, including hypertension and glomerulonephritis [3]. In our study, the mean age at KT intervention was 58.3 years in the T2DM group and 54.7 years in the non-diabetes group.

Moreover, the improvement in the age at death could be due to improvements in the overall quality of health care and increasing awareness of the disease, particularly in the population with diabetes. Although the awareness of diabetes in Korea has plateaued since 2016, it has improved in recent decades [18]. Regarding health behaviors, the rates of cigarette smoking, exercise, and excessive energy intake have also improved in the Korean population with diabetes [19].

Although ASCVD is a major cause of death in KT recipients [3,4,8], malignancy was the principal cause of death in the Korean population with diabetes undergoing KT. In the Korean population with diabetes, death from malignancy is the leading cause of sustained mortality, whereas deaths related to diabetes, cerebrovascular disease, and heart disease have decreased [10]. Death from ASCVD is common in the early phase of KT (below 1 year), and death from malignancy is more frequent later [20]. Thus, our results suggest improved longevity of KT recipients with T2DM in recent years. In addition, considering that pre-transplantation assessment includes cancer screening, these malignancies likely develop de novo. KT recipients have a 2.7-fold increased risk of death from malignancy compared to the general population; however, the proportion of individuals with a history of malignancy was less than 10% [21]. Notably, the number of pneumonia-related death was higher in our study. A decline in diabetes-related deaths was observed, but a substantial increase in death from pneumonia was observed in the population with T2DM who underwent KT, which is in accordance with previous studies that analyzed KT recipients [8]. Pneumonia is one of the most common infections after organ transplantation, accounting for 10–30% of deaths in KT recipients [22]. US data showed that diabetes increased the risk of pneumonia 1.14-fold, and ESRD due to diabetes was associated with high mortality after KT compared to other ESRD etiologies [23]. Notably, diabetes increases the risk of in KT recipients [24].

Several points remain unresolved in the current study. First, since we analyzed each individual medical record for 2 years before the date of death and defined T2DM by both diagnosis code and glucose lowering medication prescription claims, we could not verify the medication adherence and incidence of post-KT diabetes within 2 years before death. Therefore, deceased individuals with diabetes-related death in the non-diabetes group could have had post-transplantation diabetes, refused antihyperglycemic agents, or developed hyperglycemia at the time of death. Second, we could not determine the quality of life after KT because of the lack of information on cohort characteristics based on claims data. The merged data of NHIS and NSIS did not include laboratory information or anthropometric variables; thus, we were unable to determine the status of diabetes and whether hyperglycemia or diabetes-related comorbidities were controlled. Moreover, a family history of ASCVD, malignancy, and social determinants of health, including physical activity and nutritional status, which are attributed to death, were not determined. Finally, the age at the onset of diabetes and diabetes duration can impact the age at mortality in patients with T2DM undergoing KT. Unfortunately, due to the privacy policies of the NHIS, we could not access this information. Although Lim et al. have recently reported higher mortality among diabetic KT recipients < 40 years [25], our patient selection criteria were different. Suicide is the most common cause of death in the Korean population [26]; therefore, individuals who died before 30 years of age were excluded in the present study. This may have affected the included data on age at KT and at death in T2DM group.

In summary, this nationwide study of a representative sample of the total KT population indicated an improvement in age at death among KT recipients and changing causes of death in persons with and without diabetes. The age at death in KT recipients increased over the 12 years between 2006 and 2018, and this trend was similar in the diabetes and non-diabetes groups. The age at KT was higher in patients with T2DM, and was the main contributor to the age at death in KT recipients. Although our findings may reflect an improvement in the clinical prognosis of T2DM, even in patients undergoing KT, the there is still a need to control and prevent T2DM and pay attention to the population with diabetes remains. Further prospective and longitudinal studies with refined data can help further investigate and ensure complex longevity in KT recipients and the impact of controlling diabetes on the lifespan of KT recipients with T2DM.

## Figures and Tables

**Figure 3:**
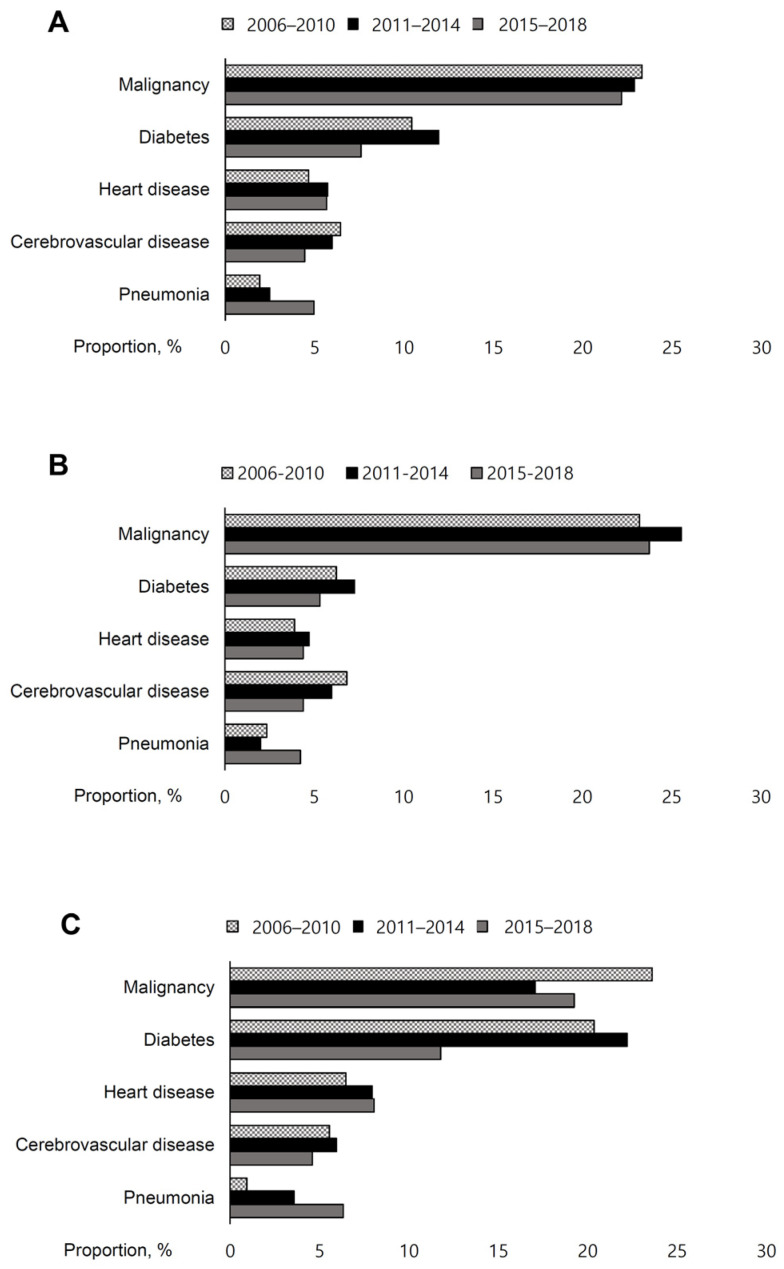
Changes in cause of death in kidney transplantation recipients. (**A**) overall population, (**B**) individuals without diabetes, (**C**) individuals with type 2 diabetes. The grey dots bars indicate 2006–2010; black solid bars, 2011–2014; grey solid bars, 2015–2018.

**Table 1:** Characteristics of deceased individuals with kidney transplantation.

	Individuals without Diabetes (*N* = 1705)	Individuals with Type 2 Diabetes (*N* = 816)	*p* Value
Age at death, years	61.5 ± 12.6	65.3 ± 10.0	<0.0001
Age groups at death, n (%)			<0.0001
30–39 years	83 (4.9)	6 (0.7)	
40–49 years	229 (13.4)	53 (6.5)	
50–59 years	422 (24.8)	144 (17.7)	
60–69 years	504 (29.6)	325 (39.8)	
70–79 years	352 (20.6)	236 (28.9)	
80+ years	115 (6.7)	52 (6.4)	
Sex, men, n (%)	1094 (64.2)	516 (63.2)	0.650
Hypertension, n (%)	1304 (76.5)	706 (86.5)	<0.0001
Years from KT to death, years	12.0 ± 3.3	11.8 ± 3.6	0.179
Age at KT, years	54.7 ± 13.0	58.3 ± 10.1	<0.0001
Cause of death			<0.0001
Malignancy, n (%)	412 (24.2)	161 (19.7)	
Cerebrovascular disease, n (%)	96 (5.6)	43 (5.3)	
Heart disease, n (%)	74 (4.3)	62 (7.6)	
Type 2 diabetes, n (%)	106 (6.2)	141 (17.3)	
Pneumonia, n (%)	50 (2.9)	33 (4.0)	
Others, n (%)	967 (56.7)	376 (46.1)	

**Table 2:** Factors influencing age at death in recipients of kidney transplantation.

Variables	Estimate (β)	Standard Error	*p* Value
Sex, women	0.037	0.017	0.033
Type 2 diabetes	0.007	0.018	0.704
Hypertension	−0.007	0.021	0.738
Age at KT	0.999	0.001	<0.001
Years from KT to death	0.971	0.002	<0.001

## Data Availability

Not applicable.

## Abbreviations

ESRD, end-stage renal disease; RRT, renal replacement therapy; KT, kidney transplantation; T2DM, type 2 diabetes; ASCVD, atherosclerotic cardiovascular disease; NHIS, Korean National Health Insurance Service; NSIS, National Statistical Information Service; BEP, benefit extension policy; SD, standard deviation; ICD, International Classification of Diseases.
